# Increase in Vascular Injury of Sodium Overloaded Mice May be Related to Vascular Angiotensin Modulation

**DOI:** 10.1371/journal.pone.0128141

**Published:** 2015-06-01

**Authors:** Cintia Taniguti Lima, Juliane Cristina de Souza Silva, Katia Aparecida da Silva Viegas, Thais Cristina de Souza Oliveira, Rariane Silva de Lima, Leandro Ezequiel de Souza, Danielle Aragão, Dulce Elena Casarini, Maria Claudia Irigoyen, Silvia Lacchini

**Affiliations:** 1 Department of Anatomy, Institute of Biomedical Sciences, University of São Paulo, São Paulo, São Paulo, Brazil; 2 Hypertension Unit, Heart Institute, University of São Paulo Medical School, São Paulo, São Paulo, Brazil; 3 Nephrology Division, Department of Medicine, Federal University of São Paulo, São Paulo, São Paulo, Brazil; Brigham and Women's Hospital, Harvard Medical School, UNITED STATES

## Abstract

This study aimed to analyzing the effect of chronic sodium overload upon carotid and femoral injury, and its relation to vascular angiotensin modulation. Male C57Bl6 mice were divided in: control (cont), receiving 1% NaCl solution for 2 weeks (salt-2) or 12 weeks (salt-12). Two-weeks before the end of the study, a 2mm catheter was implanted around the left femoral and carotid arteries to induce injury. Blood pressure (BP) and heart rate (HR) were measured at the end of the study by tail plethysmography. Arteries were collected and prepared for histological analysis to determine arterial thickening and perivascular collagen deposition. Angiotensin II and Ang(1-7) were quantified in fresh arteries using the HPLC method. There were no differences in body weight, BP and HR. Intima/media ratio had a similar increase in both injured arteries of cont and salt-2 mice, but a more pronounced increase was observed in salt-12 mice (31.1±6%). On the other hand, sodium overload modified perivascular collagen deposition, increasing thick fibers (cont: 0.5%; salt-2: 3.4%; salt-12: 0.6%) and decreasing thin fibers (cont: 7.4%; salt-2: 0.5%; salt-12: 6.8%) in non-injured arteries. Injured arteries presented similar collagen fiber distribution. Angiotensin quantification showed increased Ang(1-7) in salt treated mice (salt-2: +72%; salt-12: +45%) with a concomitant decrease in Ang II (salt-2: -54%; salt-12: -60%). Vascular injury increased significantly Ang(1-7) in salt-12 mice (+80%), maintaining Ang II reduction similar to that of a non-injured artery. The lack of changes in BP and HR suggests that the structural changes observed may be due to non-hemodynamic mechanisms such as local renin-angiotensin system. Collagen evaluation suggests that sodium overload induces time-related changes in vascular remodeling. The increase of artery injury with concomitant increase in Ang(1-7) in 12-week treated mice shows a direct association between the duration of salt treatment and the magnitude of vascular injury.

## Introduction

At an epidemiological level, it is still debatable whether a high sodium intake causes an elevation in blood pressure or increases the rate of progression into a hypertensive level [1]. Some studies in rats have reported an age-related increase in blood pressure [2] while others have not found any significant changes [3]. More recently, we have shown that high sodium intake in rats changes autonomic regulation of HR, increasing tachycardic response to arterial pressure (AP) reductions [4]. Other experimental studies in rats have found an association of salt intake with target-organ effects, such as cerebral, renal [5], and cardiac [6] arteries and respective organs. Until the last decade, it has been generally assumed that adverse cardiovascular and renal effects of increased salt intake were mediated via increase in blood pressure. Moreover, several newer experimental and clinical studies have clearly demonstrated that in addition to these pressure-independent outcomes, salt overload also exerts direct harmful effects [7]. The findings related to the effect of salt intake upon cardiovascular system have pointed hypertension as the mechanism underlying these effects.

In this context, some studies using hypertensive high salt diet- treated rats have reported arterial stiffness [8], carotid hypertrophy [9–10], and intimal lesions [11]. However, it should be emphasized that any hemodynamic modification will interfere with vascular wall. Thus, the comprehension of vascular adaptations to salt intake, regardless of blood pressure, requires a clear understanding of the role that each factor plays regardless of hemodynamic factors.

Renin-angiotensin system (RAS) is directly modulated by sodium intake, and it is closely related to RAS activity in hypertension and cardiovascular injury [12], inflammatory response [13], and to the structure of retinal arterioles [14]. On the other hand, components of RAS have been implicated in the pathogenesis of atherosclerosis in a variety of manners and as such they should be seen as important therapeutic targets. A large body of data indicates that angiotensin II (Ang II) is associated with the production of reactive oxygen species, inflammatory cytokines, and the activation of adhesion molecules [15–16]. This is also consistent with the fact that angiotensin converting enzyme (ACE) is activated in the inflammatory cells present in vascular lesions; it is also consistent with the activation of RAS components during differentiation of monocytes to macrophages [17]. ACE activity is also increased in the neointima associated with vascular injury in rats [18–19], in human carotid artery plaques [1], and in the stent-induced inflammatory mechanism [20].

There are many factors involved in the development of cardiovascular diseases; some studies have pointed the vascular structure and function derangement as a feature underlying cardiovascular diseases, and it has been associated with inflammation and endothelial activation [3–21]. Furthermore, the development of atherosclerotic lesions has as characteristic the intimal thickening which is promoted in an early essential stage of disturb. Many experimental models have been created to study this pathologic condition and to evaluate the efficacy of therapeutic strategies. The cuff-induced neointimal thickening is closely related to a well described inflammatory reaction [22–23]. In this context, we have previously shown that vascular ACE activity is directly related to neointima formation in induced vascular injury in mice [24].

Despite the relevance of potential associations described above, few reports to date have associated vascular adaptations to remodeling under sodium overload, and stated their relevance to vascular neointima formation after injury, particularly in normotension. The purpose of this study was then to evaluate whether high sodium intake can induce vascular remodeling under normotensive conditions and whether this condition may lead to vascular susceptibility to injury.

## Materials and Methods

### Animals

Experiments were performed with isogenic male C57Bl/6 mice obtained from the animal care unit of the Department of Anatomy of the Institute of Biomedical Sciences at University of São Paulo. The mice received standard laboratory chow and water *ad libitum*, and were distributed five per cage in a temperature-controlled room (22°C) with a 12-h dark-light cycle. All protocols used were in accordance with the Guidelines for Ethical Care of Experimental Animals from the International Animal Care and Use Committee. This study was approved by the University of Sao Paulo Medical School Ethical Committee (044/11). The mice were randomly assigned to one of three groups: control, receiving filtered water (Cont); 1% NaCl solution to drink for two weeks (salt-2); 1% NaCl solution to drink for twelve weeks (salt-12).

The treatments started on the 4-week old mice, and were continued until they reached 16 weeks of age. All mice have undergone surgical implantation of perivascular cuff two weeks before the end of treatments (14-weeks of age), in order to induce vascular injury and neointima formation.

### Blood Pressure Measurements

In the last week of treatment, pulse blood pressure (BP) was indirectly measured by tail plethysmography. During the week prior to the measurement, the animals were adapted to the system. BP and heart rate (HR) were measured 10 times for two consecutive days. For this, peak systolic BP were captured by the BP-2000 series II system, which consists of an electromagnetic transducer located in the tail and connected to an amplifier and to an analog-to-digital converter. To measure tail plethysmography, the mouse was placed in restraining box previously heated to promote vasodilatation in the tail thereof. The sphygmomanometer adapted to mice was placed at the tail and inflated to total obstruction of blood flow to the artery flow. The flow obstruction was decreased slowly in order to capture the first peak of systolic blood pressure.

### Femoral cuff placement

Two-weeks before the end of the study, it was performed a surgical procedure to induce femoral artery injury. To this procedure, it was used the femoral cuff placement method as described in detail previously [24]. The animals were anesthetized with an injection of Ketamine (90 mg/kg) and Xylazine (10 mg/kg) ip. Body temperature was controlled by placing the animal on operating table with heating plate to maintain the body temperature at 38°C. After isolating the left femoral artery from the surrounding tissues, a 2.0-mm polyethylene cuff (inner diameter 0.56 mm, outer diameter 0.965 mm; Becton Dickinson) was inserted around the artery, and then closed with a 4–0 cotton suture. To ensure that the blood flow was not obstructed, the cuff used was larger than the vessel. The right femoral artery did not receive the perivascular cuff, and was used as a control.

### Carotid cuff placement

Similarly as mentioned above, the surgical procedure to induce carotid artery injury was performed two-weeks before the end of the study. To this procedure, it was used the carotid cuff placement method as described in detail previously [24]. A midline incision over an area from the chin to the sternum was performed to exposure the left carotid artery, then the salivary gland was separated, and the artery was dissected proximal to the bifurcation. Thereafter, a 2.0-mm polyethylene cuff (inner diameter 0.56 mm, outer diameter 0.965 mm; Becton Dickinson) was inserted around the periphery of the carotid artery proximal to the bifurcation, and then closed with a 4–0 cotton suture. To ensure that the blood flow was not obstructed, the cuff used was larger than the vessel. Also, the right carotid artery was used as a control. After recovery from anesthesia, the animals were given a standard diet and water ad libitum.

### Tissue harvesting and histological staining

Fourteen days after implantation, mice were euthanized with an intraperitoneal injection of Ketamine (180 mg/kg) and Xylazine (20 mg/kg), received sodium heparin (50 U), and were subsequently perfused with 0.9% NaCl solution at constant pressure (80–90 mmHg) followed by a buffered 4% formalin solution. Tissues were maintained in formalin for 24–48 h to complete the fixation process, while arteries were studied using the adjacent tissues to preserve their integrity. After processing the tissues, it was embedded in paraplast for further histological analysis. It was performed different types of staining depending on the evaluation needed. To assess cellular morphology was performed Hematoxylin-Eosin staining, while to identify elastic lamina was used Verhoeff-Van Gieson staining; Picrosirius staining was used to identify collagen fiber deposition.

#### Morphometry

Morphometric analyses were performed on Verhoeff-Van Gieson, Picrossirius, and Weigert-stained tissues. Histomorphometric analyses were performed blinded to the identity of experimental groups. Five-micrometer sections were obtained every 50 μm, totaling 10 sections along the 500 μm of vessel length, corresponding to almost 25% of the total cuff-induced vascular lesion. Femoral and carotid cross sections images were digitized by computer image analysis (AxioVision 4.8 System) coupled to a Zeiss Axio Scope II microscope. For each section, the areas of the intima and media were calculated. The intimal area was calculated from the difference between the area of the inner elastic lamina and the luminal area; the area of the media layer was calculated by the difference between the area of the outer elastic lamina and the area of the inner elastic lamina. For collagen fiber deposition, slides were stained with Picrosirius and viewed by polarized light. For adequate staining slides were stained with 0.1% Sirius Red solution and counterstained with Harris's hematoxylin. Picrosirius-stained sections were evaluated by ordinary polychromatic and polarized light microscopies. Percent area of mature (red and yellow fibers) was compared to immature (green) fibers under polarized light. Elastic system fibers were studied using Weigert's resorcin-fuchsin staining. Elastic laminae were counted in 5 slices for each artery.

#### Vascular ACE immunohistochemistry

Angiotensin I converting enzyme (ACE) was measured by immunohistochemistry performed in slices of each artery stained with IgG anti-ACE antibody (Santa Cruz Biotechnology, Santa Cruz, CA). In brief, slices were deparaffinized, rehydrated, and the endogenous peroxidase activity was blocked with hydrogen peroxide (3% in water) for 20 minutes. Following rehydration, the slices were rinsed with phosphate-buffered saline (PBS). Bovine serum albumin 2% (BSA in PBS) was used to block the nonspecific sites for 60 minutes at room temperature. The primary antibody was diluted to 1:500 in TBS-TC, and applied to the sections for 16–18 hours at 4°C. Subsequently, the samples were washed and incubated with the biotinylated secondary antibody (Zymed Laboratories, South San Francisco, CA) for 60 minutes at room temperature, followed by incubation with streptavidin-peroxidase complex (1: 1000) for 60 minutes at room temperature. Finally, a 3,3’—diaminobenzidine solution (DAB, Vector Labs.) was applied. The slices were coded and then assessed by two independent blinded observers using an optical microscope (Zeiss Axio Scope II) equipped with a 40x objective and coupled to an image analyzer (Axio Vision 4.8 System).

### Angiotensin quantification

For angiotensin vascular quantification, the groups were prepared and their arteries were collected. The animals were anesthetized (Ketamine 90mg/kg and 10mg/kg Xylazine, ip) and fragments of femoral arteries were collected. The mice were subsequently euthanatized by an overdose of the same anesthetic and the carotid arteries were removed. To harvest control vessels (without injury) we collected approximately 2mm segments from the region corresponding to that of vascular injury (in the contralateral vessel), while for the arteries with injury, we collected a 2mm fragment surrounded by cuff. Tissues were removed, immediately frozen and kept at -80^°^C until measurement. These femoral and carotid arteries were used for quantification of angiotensin I, II and 1–7 [25]. Angiotensin extraction was performed using in Sep-Pak-C18 columns (Waters, Milford, MA), and then, peptides were measured by HPLC, as described by Almeida et al., 2006 [26]. Peptides were identified according to retention time. The identity of eluted ANG I, ANG II, and ANG 1–7 was confirmed by direct sequencing (protein sequencer PPSQ-23; Shimadzu, Tokyo, Japan). For concentration determination, commercially available peptides were employed to develop a standard curve. Peptide levels were expressed as pictograms per artery, as described in a previous study [24]. These were not normalized according to total protein concentration since total protein was at very low concentrations.

### Statistical analysis

All values are expressed as means ± SD. Morphometric evaluations were first tested by two-way ANOVA, while angiotensins were tested by one-way ANOVA. When ANOVA demonstrated significant differences, Tukey's post hoc analysis was used to compare groups. For all statistical analyses, *P* ≤ 0.05 was considered statistically significant.

## Results

### Body weight and hemodynamic measurements

**Fig 1:**
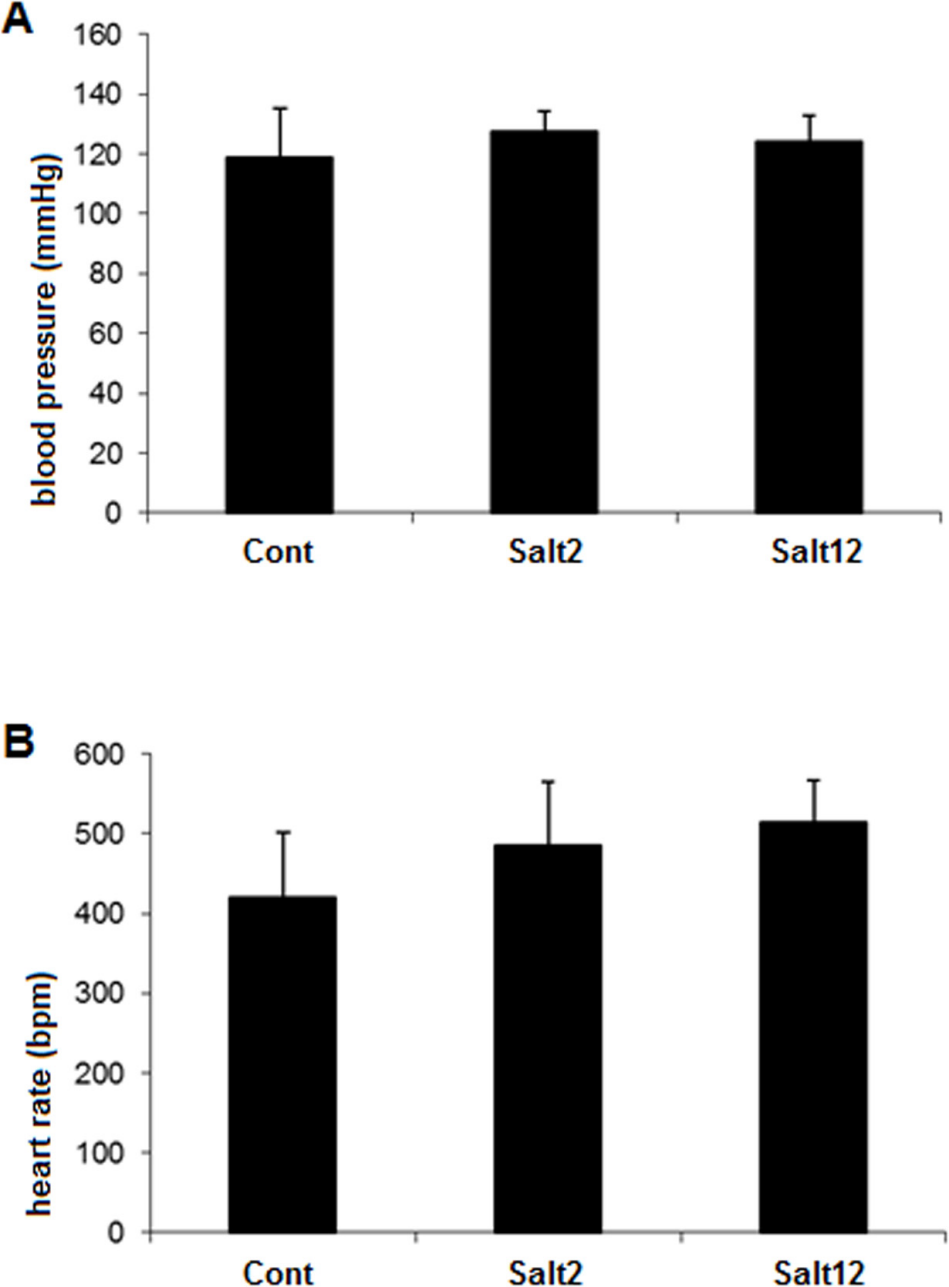
Hemodynamic measurements. Systolic blood pressure (1*A*) and heart rate (1*B*) assessed by tail plethysmography. As can be observed, there was no difference between the groups.

Body weight at the start and at the end of protocol was similar between the three groups. Indirect measurements showed that blood pressure (Fig 1*A*) and heart rate levels (Fig 1*B*) remained essentially the same in all groups regardless of the high salt treatments (S1 Table).

### Neointima thickening in femoral and carotid arteries

**Fig 2:**
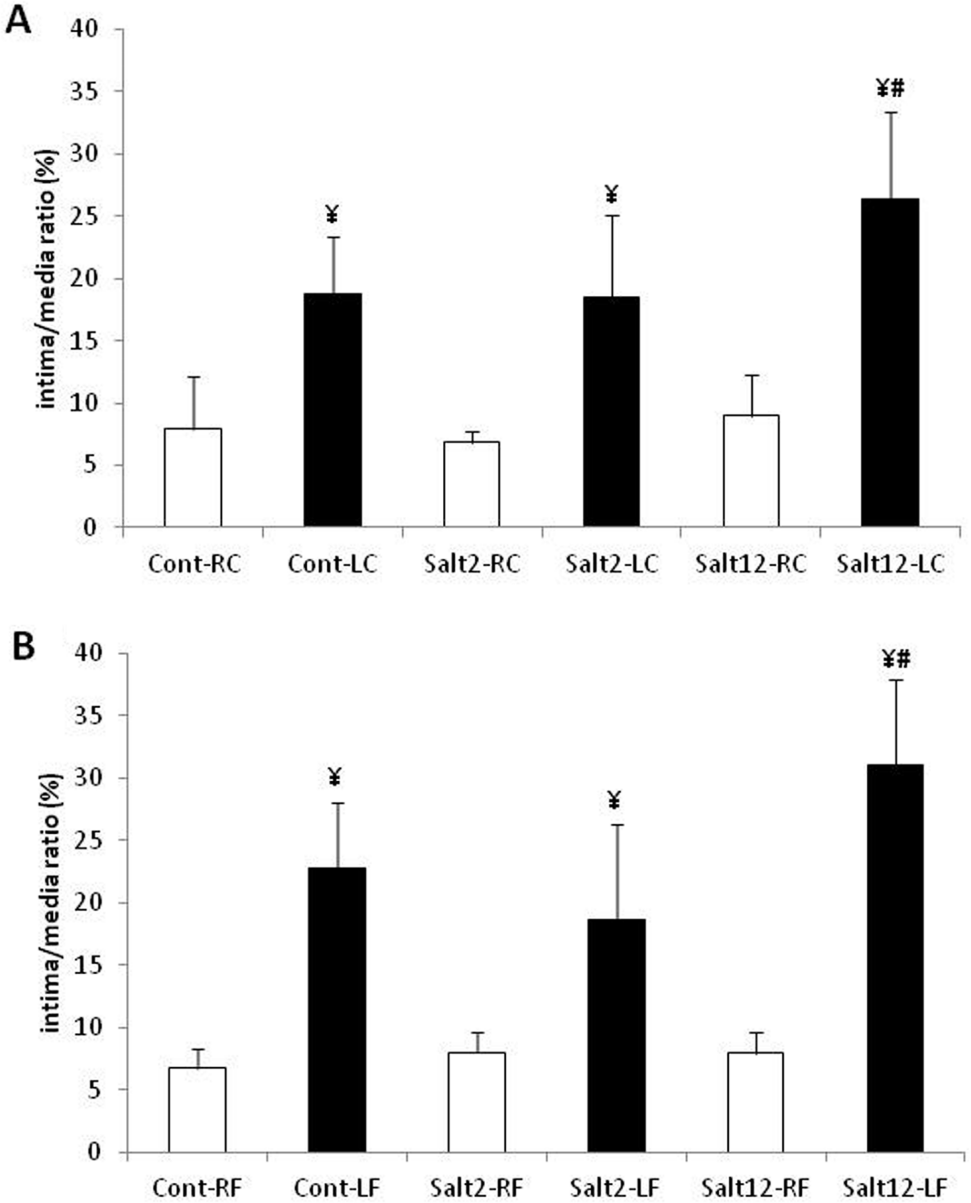
Intima/media ratio in femoral and carotid arteries. A: carotid artery, and B: femoral artery. Observe the increase in I/M ratio after vascular injury in all treatments. White bar: control, non-injured artery; black bar: injured artery. ¥ p<0.05 when compared to non-injured artery; # p<0.05 when compared to injured artery of cont and salt2 groups.

Upon cuff-induced injury, all mice developed vascular neointima thickening regardless of either fresh water or salt intake. Fig 2*A* shows the relative area of neointima, comparing non-injured and injured carotid arteries in control and in two and twelve-week treated mice. The neointima-to-media ratio showed a significant increase in cont and salt-2 groups, and a greater increase in salt-12 mice. Fig 2*B* also shows that in femoral arteries the neointima-to-media ratio cont and salt-2 groups, with a larger increase in the salt-12 group (S2 Table). The area of the media was not different among the four groups regardless of neointima formation (data not shown), thus indicating an injury process without vascular remodeling.

### Elastic lamellae analysis

**Fig 3:**
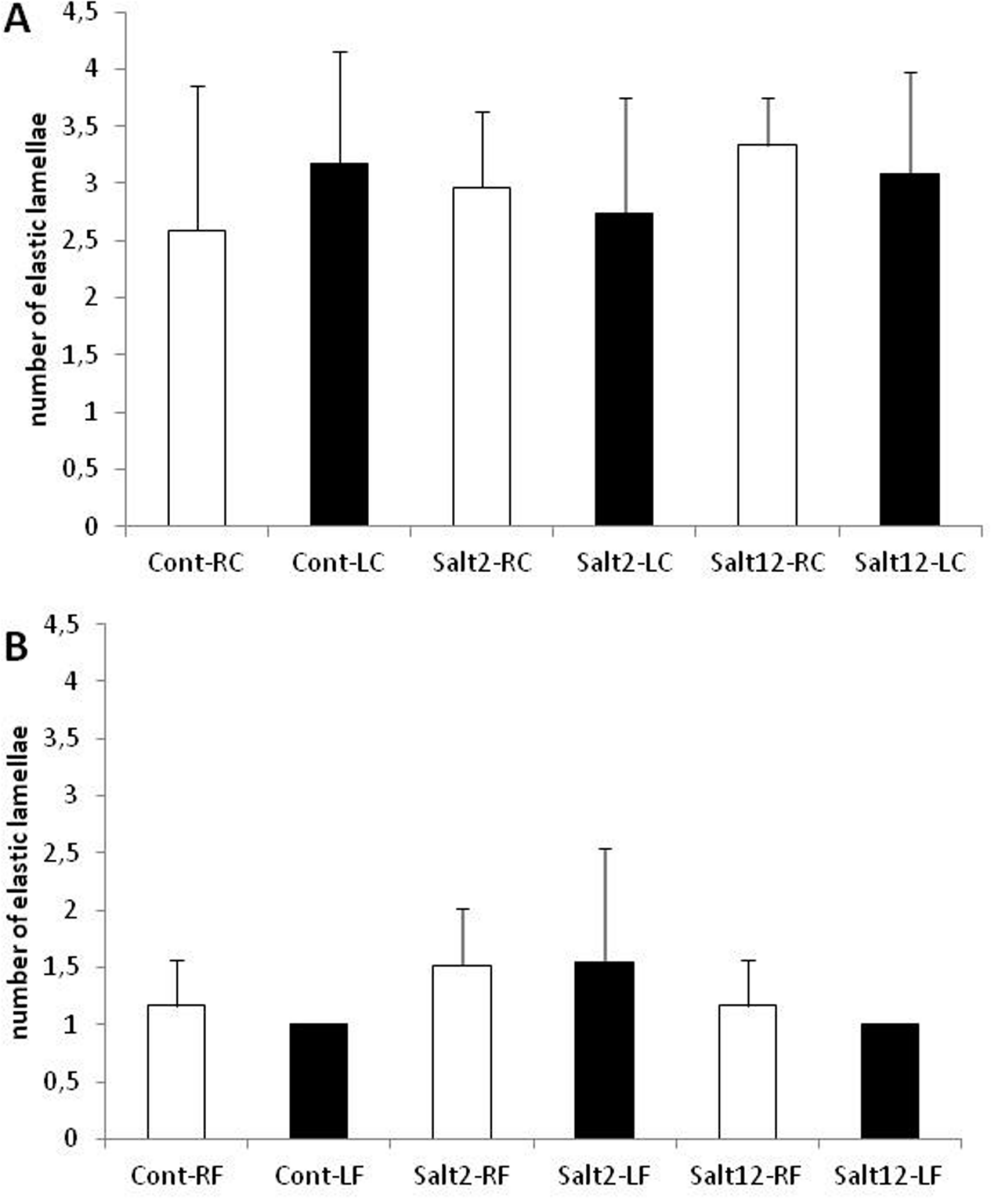
Number of elastic lamellae in femoral and carotid arteries. A: carotid artery, and B: femoral artery. White bar: control, non-injured artery; black bar: injured artery. No differences were observed between treatments.

Fig 3 presents the counting of elastic lamellae in carotid and femoral arteries. As can be observed, there were no changes in this elastic component associated with salt intake. Also, vascular injury did not change lamellae distribution in femoral and carotid arteries.

### Collagen fibers deposition

**Fig 4:**
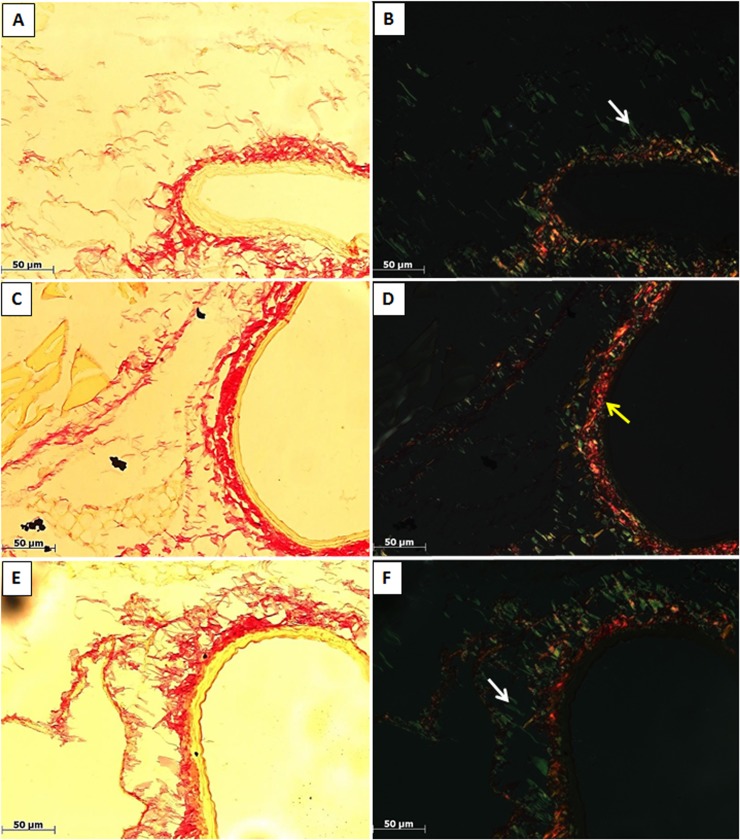
Vascular collagen fiber deposition. Picrosirius red-stained sections assessed by bright field (A, C, E—left column) and polarized light (B, D, E—right column), indicate perivascular fibrosis in carotid arteries. The polarized light showed an important deposition of thin, young collagen fibers (green), a relative low deposition of mature (thick, red) and intermediary (yellow) collagen fibers in control group. On the other hand, salt2 group showed a significant reduction of thin fibers and increase of intermediary and thick collagen fibers. Salt12 group showed a pattern similar to the control group. (Magnification 1000x. Groups: Control, A-B; Salt2, C-D; Salt12, E-F. White arrow: thin fiber; Yellow arrow: thick fibers).

Collagen fibers were studied under polarized light, and the percent measures of thin and thick fibers are shown in Table 1. The vascular collagen fiber deposition was similar in femoral and carotid arteries of control mice, both in non-injured carotid and femoral arteries and in injured carotid and femoral arteries. In line with this pattern, an important deposition of thin, young fiber (in green color), was found, together with a relative low deposition of mature (red) and intermediary (yellow) fibers (Fig 4). Interestingly, 2-weeks of salt intake changed this pattern, with an important reduction of young fibers, and increase in intermediary and mature collagen fibers. This response was found for both non-injured carotid and femoral arteries and for injured carotid and femoral arteries. On the other hand, a 12-week salt intake showed a return to a pattern similar to that of control mice. However, after 12-weeks of salt intake the injured carotid presented an intense deposition of all mature, intermediary and young collagen fibers.

**Table 1:** Percent values of collagen fibers composition.

***Femoral artery***			
		***without injury***	***injury***
Cont	Thin fibers	89.41 ± 6.57	90.17 ± 3.10
	Thick fibers	10.59 ±6.57	9.83 ± 3.10
Salt2	Thin fibers	3.77 ± 2.71 *	3.417 ± 1.63
	Thick fibers	96.23 ± 2.71 ¥	96.59 ± 1.63
Salt12	Thin fibers	80.16 ± 6.64	88.09 ± 8.90
	Thick fibers	19.84 ± 6.64 ¥	11.91 ±. 8.90 **
***Carotid artery***			
		***without injury***	***injury***
Cont	Thin fibers	71.21 ± 9.63	75.07 ± 7.92
	Thick fibers	28.79 ± 9.63	24.93 ± 7.92
Salt2	Thin fibers	8.85 ± 6.06 *	6.72 ± 5.28
	Thick fibers	91.15 ± 6.06 ¥	93.28 ± 5.28
Salt12	Thin fibers	71.21 ± 16.17	70.70 ± 8.70
	Thick fibers	28.79 ± 16.17	29.30 ± 8.70

Red: thick fibers; green: thin fibers; yellow: intermediary fibers.

* p≤0.05 compared to thin fibers of control group

¥ p≤0.05 compared to thick fibers of control group

** p≤0.05 compared to non-injured artery.

### ACE immunohistochemistry

**Fig 5:**
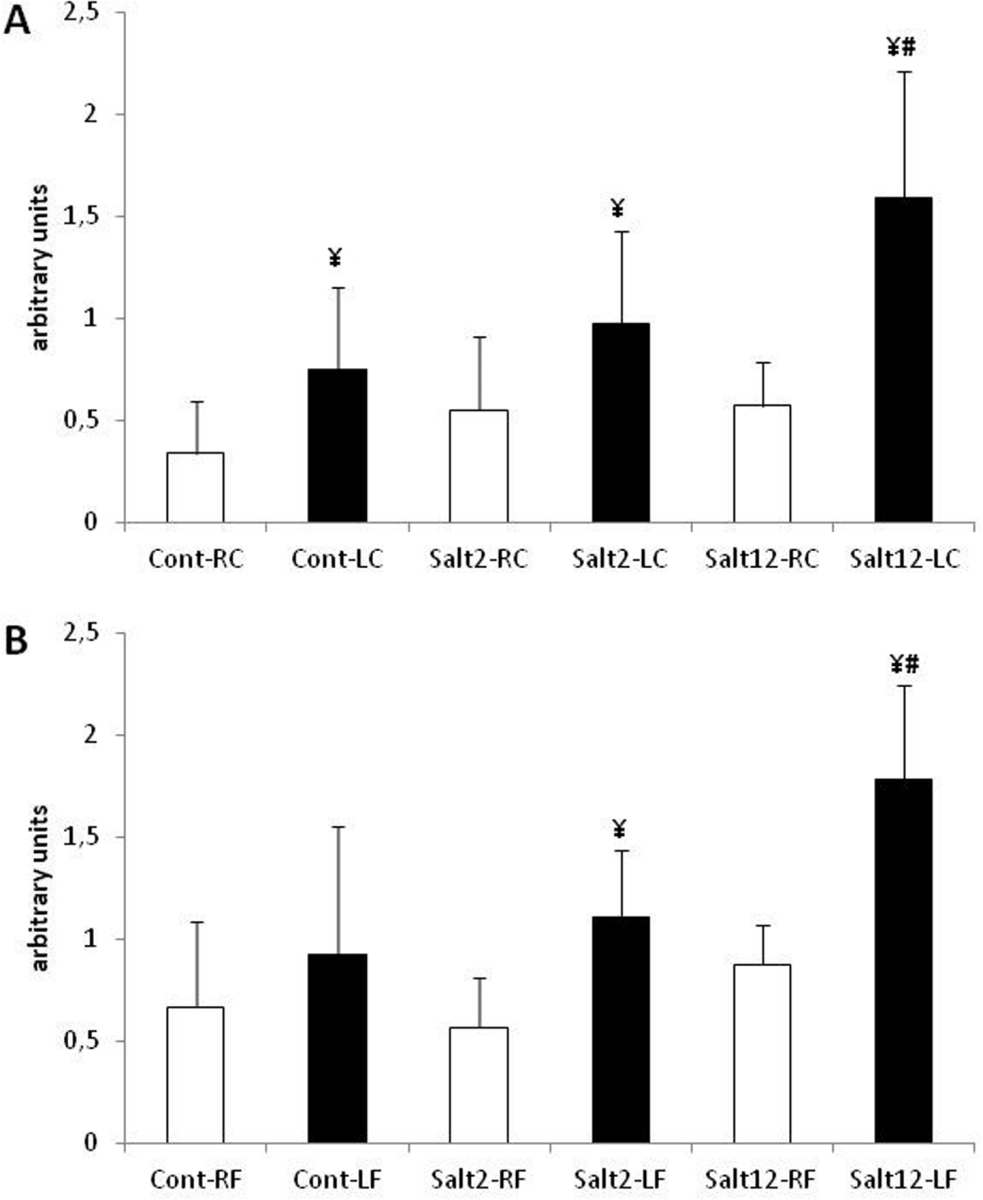
Score evaluation of ACE immunohistochemistry in femoral and carotid arteries. A: carotid artery, and B: femoral artery. Observe the increase of staining after vascular injury in all treatments. White bar: control, non-injured artery; black bar: injured artery. ¥ p<0.05 compared to non-injured artery; # p<0.05 compared to injured artery of cont and salt2 groups.

ACE was evaluated by the staining score in histological prepared vessels. Fig 5 presents a semi-quantitative analysis of this immunohistochemistry in carotid (5*A*) and femoral (5*B*) arteries. The results were similar to those obtained for intima/media ratio, when all injured vessels presented increases in antibody staining, with a larger increase found for 12-week salt treated mice.

### Angiotensin evaluation

**Fig 6:**
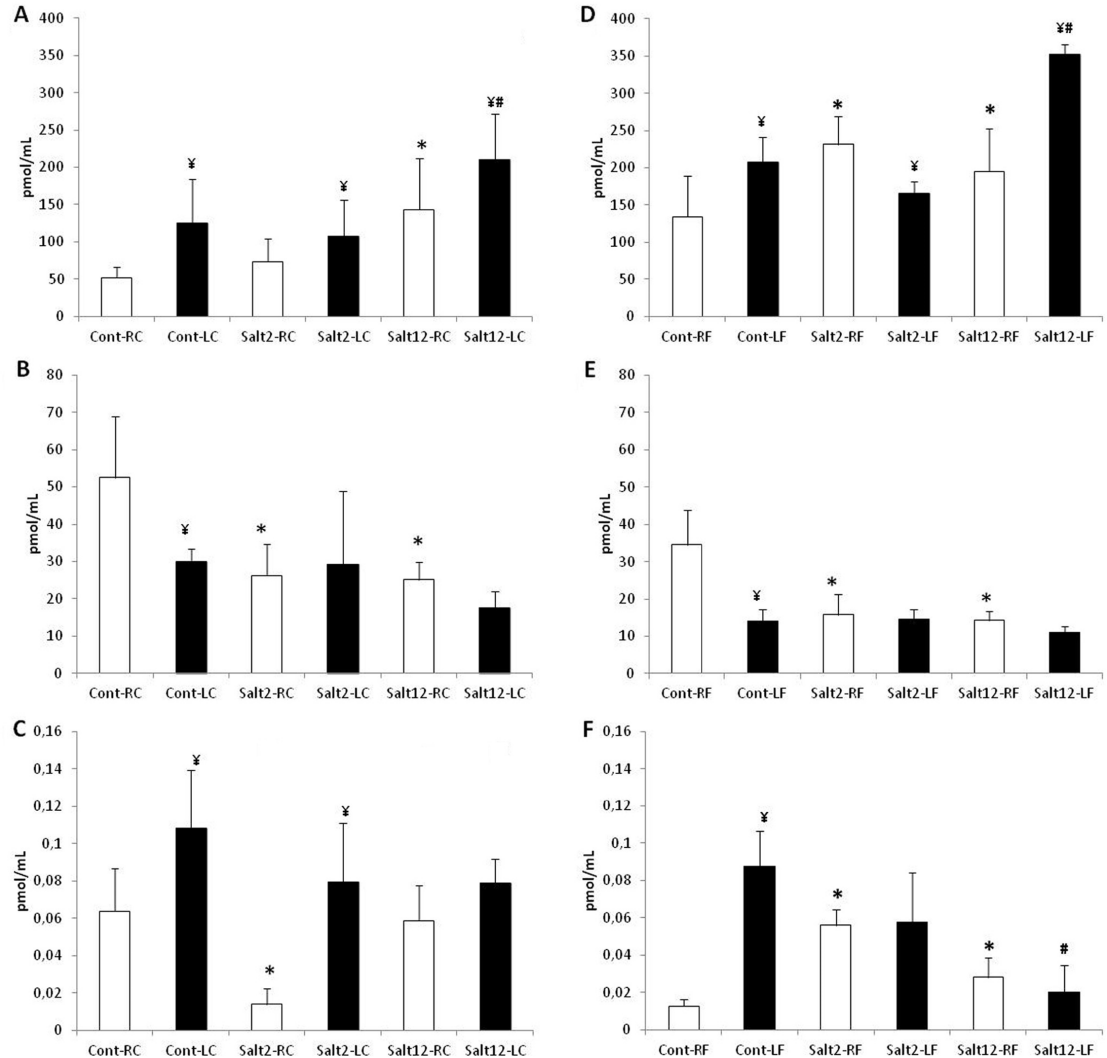
Vascular quantification of angiotensin in femoral and carotid arteries. A: Ang (1–7) quantification in carotid artery, B: Ang II quantification in carotid artery, C: Ang I quantification in carotid artery, D: Ang (1–7) quantification in femoral artery, E: Ang II quantification in femoral artery, F: Ang I quantification in femoral artery. Observe the increase in I/M ratio after vascular injury in all treatments. White bar: control, non-injured artery; black bar: injured artery. * p<0.05 compared to control group; ¥ p<0.05 compared to non-injured artery; # p<0.05 compared to injured artery of cont and salt2 groups.

Angiotensin I, II and (1–7) were measured in 2mm of carotid and femoral arteries, and the results obtained are presented in Fig 6. It can be observed that in carotid artery (6*A*, 6*B* and 6*C*) salt intake increases Ang (1–7) and decreases Ang II, with low interference in Ang I. However, after injury, we did not observe Ang II reduction in salt-2 and salt-12 mice with a concomitant reduction of Ang I. A similar response was found for femoral arteries (6*D*, 6*E* and 6*F*).

## Discussion

This study assessed the effect of salt overload upon femoral and carotid injury in mice. The main findings of the present study were that high salt intake increases the magnitude of vascular injury without blood pressure increments. In addition, high salt intake interferes with vascular collagen deposition and Ang II and Ang (1–7) synthesis, which may be associated with vascular injury.

We observed that high salt treatment did not change hemodynamic parameters, such as arterial pressure and heart rate. These findings are consistent with previous studies in rats [24] and mice [27], showing that there was no blood pressure increase in normotensive animals receiving 1% saline to drink.

Many clinical and experimental studies have associated high salt consumption to blood pressure increase [28]. However, other studies have suggested that a high salt intake may be directly associated with the risk of stroke, left ventricular hypertrophy, and other conditions without increments on blood pressure [29,30,31].

### Femoral and carotid arteries morphometry

The tunica intima/ media thickness ratio has been widely used to quantify the magnitude of vascular injury, as it clearly informs the neointimal response after injury. Moreover, it is well established that implantation of a perivascular catheter is capable of inducing a local inflammatory reaction, which triggers neointima formation. Although we have not observed changes in blood pressure, we might speculate that salt overload indicates that some sort of adjustment must have influenced the magnitude of this lesion. The difference in injury magnitude measured in animals undergoing 12 weeks of salt overload suggests that there is some kind of imbalance of regulatory factors. Clinical studies have shown that salt-sensitive patients display increased inflammatory markers, such as p-selectin, e-selectin and monocyte chemotactic protein type 1 (MCP-1) when compared to salt-resistant ones [32]. Moreover, experimental observations in normotensive rats have found that sodium loading promotes neointimal formation, even in normotensive rats, and that hypertension further stimulates this neointimal formation [11–33].

Carotid and femoral quantification of elastic lamellae by Weigert’s staining showed no change in the treated groups when compared to control, both in the carotid arteries and in the femoral arteries. The fact that we did not measure the differences between treatments suggests that the stimulus offered by the treatments was not enough to determine a significant change in the elastic system. However, a more detailed assessment, which would include a closer look at elastic fibers, may find whether salt overload effectively alters the elastic system.

On the other hand, the analysis of collagen fibers in both femoral and carotid arteries shows a percent distribution with a predominance of less mature thin fibers, thus suggesting a constant renewal of collagen system, a pattern that would hold after vascular injury. However, salt overload treatments seem to alter the synthesis, degradation and/or deposition of collagen fibers, since after 2 weeks of salt overload there is a substantial reduction of thin fibers (possibly along with reduced synthesis), while intermediate fibers (more mature) are increased, probably due to a reduction in the degradation of the existing fibers. Another point worth mentioning lies in the fact that in the more chronically treated mice with saline, the distribution pattern of collagen fibers closely resembles that of the control group.

Interestingly, the injury did not modify this collagen composition in femoral and carotid arteries. However, since the thin-thick fiber ratio in femoral artery of 12-week treated group is similar to control group, the number of thick fibers increases, thus suggesting an arterial stiffening process. The percent distribution of collagen fibers in the femoral arteries showed similar results to those observed in the carotid arteries, for which there is a different distribution of collagen fibers in the group receiving saline overload for 2 weeks. The increase in collagen deposition was previously described in rats [34]. In that study, a 1% saline solution promoted carotid collagen deposition in stroke-prone spontaneously hypertensive rats [34], as well intimal proliferations and necrotic lesions in SHR intrarenal arteries [11] or associated to cardiac fibrosis in SHR [35]. These findings offer a very interesting perspective, suggesting that the collagen system is sensitive to the amount of salt intake, and may indeed play a role in the mechanism of arterial stiffening. However, we should not neglect the hypertension-related findings of those previous studies. Since hypertension increases inflammatory markers which may contribute to hypertension “per se”, to collagen deposition and vascular injury, the present study reinforces the argument that some processes may take place in the vascular wall, regardless of increases in blood pressure.

### Effect on renin-angiotensin system

It should be noted that the intensity of ACE staining by immunohistochemistry corresponds to the intensity of vascular injury observed in both carotid and femoral arteries. In a previous study, we described the relationship between the increase of Ace gene copies, ACE vascular activity and vascular injury in genetic modified mice [24]. Also, we found that vascular injury is related to an increase in the ACE staining in all groups; however, mice receiving saline overload for 12 weeks showed a more intense staining than the other groups. On the other hand, the quantification of vascular angiotensin by HPLC lends support to the results obtained in the immunohistochemistry study. We found that both femoral and carotid arteries showed a change in the Ang II—Ang (1–7) ratio in animals receiving saline overload. In these groups, we found a significant reduction of Ang II and progressive increase in Ang (1–7), and this may work as a protective mechanism in vascular physiological conditions, possibly by altering the activity of enzymes forming Ang II (reduced ACE) and Ang (1–7) (increased ACE2).

Although we have not evaluated the activities of these enzymes, two of our findings seem to lend support to this argument: 1) we did not find any change in Ang I in these same vessels, suggesting that ACE2 may be cleaving Ang II to Ang (1–7), and 2) the positive staining for ACE in immunohistochemistry in the carotid without lesion; although it cannot demonstrate the activity of this enzyme per se, it may very well be related to an unchanged regulation. In addition, the evaluation of the carotid and femoral arteries undergoing vascular injury showed that the increase in Ang (1–7) observed in animals which received salt overload persisted for 12 weeks.

Recently, Mas receptor of Ang (1–7) [35], and plasma renin activity [36] have been associated with salt-sensitive hypertension. Previous studies have shown a reduction of cardiac Ang (1–7) associated to incapacity to control hypertension in spontaneously hypertensive rats (SHR) receiving high sodium intake [37]. However, similar to our results, Roks and coworkers (2004), also found a modulation of Ang (1–7) function through increased sodium intake in normotensive rats [38]. In this sense, a high sodium intake increases the contribution of Ang (1–7) for the maintenance of blood pressure in normotensive models. Considering these results, we may suggest that Ang (1–7) production along with Mas receptor synthesis serves as an endogenous feedback mechanism against Ang II, and would be related to a vascular adaptation to high salt intake.

On the other hands, it should be emphasized that further reductions of Ang II in injured arteries of mice with saline overload were not detected and that these vessels might have increased ACE activity, as suggested by both reduced Ang I and increased positive staining for ACE in immunohistochemistry. In this context, this Ang II amount should be directly related to the increased of vascular injury. It is well established that Ang II leads to vascular smooth muscle cells migration, extracellular matrix deposition and vascular remodeling [39], and the AT1 receptor blockade prevents vascular lesion [40]. The results observed suggest that this non-reduced Ang II in cuff injured arteries leads to the observed increase in intima/media ratio, characterizing the increased vascular lesion.

Collectively, our data suggests that 1% saline intake increases neointima formation after injury and vascular collagen deposition. This increase in neointima formation is probably related to local interaction between angiotensin II and angiotensin (1–7).

## Supporting Information

S1 Table Hemodynamic values obtained in Cont, Salt2 and Salt12 groups. Systolic blood Pressure (SBP) and heart hate were determined in control (cont), Salt2 and Salt12. Data are means ± SDM.
(PDF)

S2 Table Morphometric and biochemical results. Intima-to-media ratio, elastic lamellae and biochemical analysis of ACE, Ang I, Ang II and Ang-(1–7) were determined in control (cont), Salt2 and Salt12. LC: left carotid artery; RC: right carotid artery; LF: left femoral artery; RF: right femoral artery. Data are means ± SDM.
(PDF)
