# Supplementary material for: Increase in Vascular Injury of Sodium Overloaded Mice May be Related to Vascular Angiotensin Modulation
Source: PLoS One. 2015 Jun 1;10(6):e0128141. doi: 10.1371/journal.pone.0128141 (PMC4451144; doi:10.1371/journal.pone.0128141)
Supplement: S1 Table — Systolic blood Pressure (SBP) and heart hate were determined in control (cont), Salt2 and Salt12. Data are means ± SDM. (PDF) [file pone.0128141.s001.pdf]

S1 Table. Hemodynamic values obtained in Cont, Salt2 and Salt12 groups

	SBP (mmHg)	HR (bpm)
Cont	119.35 ± 14.08	470 ± 77.44
Salt2	127.68 ± 6.70	486 ± 79.59
Salt12	124.07 ± 8.64	524 ± 52.65

Systolic blood Pressure (SBP) and heart hate were determined in control (cont), Salt2 and Salt12. Data are means ± SDM.
