# Supplementary material for: Increase in Vascular Injury of Sodium Overloaded Mice May be Related to Vascular Angiotensin Modulation
Source: PLoS One. 2015 Jun 1;10(6):e0128141. doi: 10.1371/journal.pone.0128141 (PMC4451144; doi:10.1371/journal.pone.0128141)
Supplement: S2 Table — Intima-to-media ratio, elastic lamellae and biochemical analysis of ACE, Ang I, Ang II and Ang-(1–7) were determined in control (cont), Salt2 and Salt12. LC: left carotid artery; RC: right carotid artery; LF: left femoral artery; RF: right femoral artery. Data are means ± SDM. (PDF) [file pone.0128141.s002.pdf]

S2 Table. Morphometric and biochemical results

		Intima/Media ratio (%)	Elastic Lamellae (number)	ACE (arbitrary units)	Ang I (pmol/ml)	Ang II (pmol/ml)	Ang-(1-7) (pmol/ml)
Cont	LC	18.73 ± 4.64	3.16 ± 0.99	0.65 ± 0.45	0.10 ± 0.03	30.09 ± 3.35	124.59 ± 59.29
	RC	7.92 ± 4.19	2.59 ± 1.25	0.33 ± 0.25	0.06 ± 0.02	52.47 ± 16.42	51.72 ± 14.98
	LF	22.75 ± 5.34	1.00 ± 0.00	1.20 ± 0.95	0.08 ± 0.01	13.94 ± 3.22	208.17 ± 32.94
	RF	6.77 ± 1.57	1.16 ± 0.40	0.66 ± 0.42	0.01 ± 0.00	34.50 ± 9.31	134.42 ± 55.06
Salt2	LC	18.49 ± 6.56	3.11 ± 1.01	0.56 ± 0.53	0.07 ± 0.03	29.22 ± 19.74	107.85 ± 47.90
	RC	6.85 ± 0.84	2.96 ± 0.65	0.55 ± 0.35	0.01 ± 0.00	26.19 ± 8.57	73.36 ± 30.47
	LF	18.60 ± 7.68	1.54 ± 1.00	1.10 ± 0.32	0.05 ± 0.02	14.53 ± 2.57	165.91 ± 15.36
	RF	8.03 ± 1.63	1.51 ± 0.50	0.56 ± 0.24	0.05 ± 0.00	15.76 ± 5.40	231.45 ± 37.06
Salt12	LC	26.33 ± 6.98	3.07 ± 0.89	1.58 ± 0.62	0.07 ± 0.01	17.53 ± 4.57	210.16 ± 61.26
	RC	8.97 ± 3.35	3.33 ± 0.41	0.93 ± 0.81	0.05 ± 0.01	25.04 ± 4.81	143.02 ± 68.86
	LF	31.05 ± 6.92	1.00 ± 0.00	1.67 ± 0.53	0.02 ± 0.01	11.07 ± 1.49	352.26 ± 13.40
	RF	7.97 ± 1.69	1.16 ± 0.40	1.12 ± 0.33	0.02 ± 0.01	14.26 ± 2.39	195.13 ± 58.13

Intima-to-media ratio, elastic lamellae and biochemical analysis of ACE, Ang I, Ang II and Ang-(1-7) were determined in control (cont), Salt2 and Salt12. LC: left carotid artery; RC: right carotid artery; LF: left femoral artery; RF: right femoral artery. Data are means ± SDM.
